# Should Airway Interstitial Fluid Be Used to Evaluate the Pharmacokinetics of Macrolide Antibiotics for Dose Regimen Determination in Respiratory Infection?

**DOI:** 10.3390/antibiotics12040700

**Published:** 2023-04-03

**Authors:** Jianzhong Wang, Xueying Zhou, Sara T. Elazab, Seung-Chun Park, Walter H. Hsu

**Affiliations:** 1 Shanxi Key Laboratory for Modernization of TCVM, College of Veterinary Medicine, Shanxi Agricultural University, Taigu, Jinzhong 030810, China; 2 Department of Veterinary Clinical Science, College of Veterinary Medicine, China Agricultural University, Beijing 100107, China; 3 Department of Pharmacology, Faculty of Veterinary Medicine, Mansoura University, El-Mansoura 35516, Egypt; 4 Laboratory of Veterinary Pharmacokinetics and Pharmacodynamics, College of Veterinary Medicine, Kyungpook National University, Daegu 41566, Republic of Korea; 5 Department of Biomedical Sciences, College of Veterinary Medicine, Iowa State University, Ames, IA 50011-2042, USA

**Keywords:** serum/plasma concentrations, interstitial concentrations, tissue concentrations, pharmacokinetics, macrolide antibiotics

## Abstract

Macrolide antibiotics are important drugs to combat infections. The pharmacokinetics (PK) of these drugs are essential for the determination of their optimal dose regimens, which affect antimicrobial pharmacodynamics and treatment success. For most drugs, the measurement of their concentrations in plasma/serum is the surrogate for drug concentrations in target tissues for therapy. However, for macrolides, simple reliance on total or free drug concentrations in serum/plasma might be misleading. The macrolide antibiotic concentrations of serum/plasma, interstitial fluid (ISF), and target tissue itself usually yield very different PK results. In fact, the PK of a macrolide antibiotic based on serum/plasma concentrations alone is not an ideal predictor for the in vivo efficacy against respiratory pathogens. Instead, the PK based on drug concentrations at the site of infection or ISF provide much more clinically relevant information than serum/plasma concentrations. This review aims to summarize and compare/discuss the use of drug concentrations of serum/plasma, airway ISF, and tissues for computing the PK of macrolides. A better understanding of the PK of macrolide antibiotics based on airway ISF concentrations will help optimize the antibacterial dose regimen as well as minimizing toxicity and the emergence of drug resistance in clinical practice.

## 1. Introduction

Macrolide antibiotics are a family of compounds featured by the existence of a macrocyclic lactone ring of ≥12 members [1,2]. The macrolide molecule is hydrophobic and is distributed in the extracellular fluid. In consideration of their satisfactory bioavailability via oral administration, superior tissue penetration and broad efficacy against many pulmonary pathogens, macrolides are extensively used as first-line antibiotics for the treatment of respiratory bacterial infections [3].

Pharmacokinetics (PK) describe the chronological movement of drugs within the body, i.e., the time course of the drug concentrations of serum/plasma or tissue fluid. Understanding the PK plays an important role in monitoring the antibiotic exposure in a patient. PK are most frequently evaluated by measuring the drug concentrations of serum/plasma. In addition to PK, the optimal dosing of an antibiotic also relies on the pharmacodynamics (PD) of the drug. The PD of a drug describe the relationship between drug concentration and pharmacological activity. Usually, the measurement of drug exposure is based on serum/plasma concentration–time course data [4]. Traditional PD are based on serum/plasma concentrations of a drug which achieve equilibrium with tissues. The selection of antibiotic concentrations aims to obtain an ideal exposure that should maximize the antibacterial activity and minimize antibiotic resistance [4,5,6,7]. Thus, most investigators have focused on the drug concentrations of serum/plasma for the PK/PD studies. However, the interstitial tissue is a site invaded by most bacteria [8]. The concentration of an antibiotic in the interstitial fluid (ISF) of the target tissue is essential for assessing the antibacterial effects [8]. Therefore, researchers realize that only the free (unbound) antibiotic concentration in the ISF of the target site is in charge of the antibacterial activity and is more applicable in determining clinical efficacy than serum/plasma concentration [9,10,11,12]. However, for most antibiotics, serum/plasma concentrations have been used to calculate PK/PD for the determination of the optimal dose regimens [11]. Meanwhile, the traditional PD parameters based on the serum/plasma concentration of a macrolide antibiotic are not suitable for the management of respiratory infections due to their much higher concentrations in the respiratory tract than serum/plasma [4]. Plasma/serum concentrations of macrolide antibiotics (e.g., clarithromycin, tildipirosin, gamithromycin, tilmicosin, telithromycin, and tulathromycin) in animals following the administration of recommended doses are, overall, substantially lower than their minimum inhibitory concentrations (MICs) [13]. The aforementioned studies indicated that the length of time when the drug concentration remains higher than MIC of the pathogen at the infection site, which provides more therapeutically relevant data than depending on serum/plasma concentrations [14].

This review aims to address the use of serum/plasma concentrations and pulmonary tissue and ISF concentrations for the PK/PD of macrolide antibiotics.

## 2. ISF Concentrations of Macrolide Antibiotics in the Lower Respiratory Tract

For most drugs in general, the measurement of their concentrations in the serum/plasma is the surrogate for drug concentrations in target tissues for therapy [4]. However, most infections appear in tissues instead of the blood [15]. There is increasing interest in the relationship between the PK and PD of antimicrobial agents [11,16,17], such as MIC, C_max_/MIC, and AUC_24_/MIC, which rely on the serum/plasma concentration as the PK input value and MIC as the PD input value [9]. Further understanding of PK and PD is attainable via meticulous PK investigations, the use of the free drug concentration of serum/plasma in confirming PK values, and the analysis of drug concentrations of tissues and ISF [18]. The precise assessment of the drug concentration at the infection site is necessary for the optimal therapy in patients. Measurements in special compartments (such as epithelial lining fluid) or confirming concentrations in ISF contributes to our understanding of macrolide concentrations at the infection site, thereby leading to the high therapeutic efficacy of macrolides. Following a macrolide administration, different tissues may contain different macrolide concentrations. In a mouse model, it was found that after clarithromycin administration to *Streptococcus pneumoniae*-inoculated mice, the incubation of lung and thigh tissues yielded very different bacteria counts: a drastic reduction in the lung and an increase in the thigh [19]. Thus, consistent bacterial killing was observed in the lung model of infection whereas no drug effect was seen in the thigh model. These results implied that following the macrolide administration, its concentration is tissue-dependent. The lungs may have a much higher macrolide concentration than the thigh. Others also reported that lung tissues contain higher macrolide concentrations than those of muscle [20], fat [21], and skin [22]. In addition, higher clarithromycin concentrations were found in pulmonary epithelial lining fluid (PELF) than the serum of mice [19]. In another mouse study, ~19-fold higher clarithromycin concentrations were found in lungs than plasma following a 100 mg/kg clarithromycin dose [23]. The unique characteristic of macrolide antibiotics with a higher concentration in lungs and PELF than serum/plasma is an exciting phenomenon.

A series of physicochemical factors might facilitate the pulmonary preference of macrolides, including low molecular weight and an extensive level of dissociation/ionization at the plasma pH of 7.4 [24,25]. According to the supposable concept of ion trapping, basic/ionized chemicals are easily trapped in the acidic environment of organelles after being transported into cells [26,27]. Macrolides are chemically lipophilic and basic (PK_a_ > 7), with a low molecular weight of <1 KDa [28]. Normal airway surface liquid and alveolar subphase fluid (pH of 6.92 ± 0.01) are both more acidic than plasma [29]. The presence of pneumonia in bronchi was associated with a lower pH than that in noninfected bronchi: 6.48 ± 0.12 vs. 6.69 ± 0.13 (*p* < 0.05) [30]. The low degree of ionization under the plasma pH is likely to promote a higher drug concentration in acidic sites, e.g., the PELF of patients with pneumonia [29,31]. Therefore, the higher lung concentrations of macrolide antibiotics may yield higher clinical efficacy. However, a higher local drug concentration is not always consistent with the clinical efficacy, as local situations might be an influential factor of drug activity. Erythromycin may be accumulated markedly in an acidic site, but it is unstable at a low pH and may turn into a dysfunctional molecule in an acidic environment [32]. In addition, the capability of drug penetration into the extravascular space is influenced by a number of factors, including the extent of plasma protein and tissue binding, the drug’s molecular charge and size as well as lipid solubility, and blood flow at the site of infection [15,33]. Macrolide antibiotics are basic compounds, poorly soluble in water, which are mostly absorbed in the alkaline intestinal environment [34]. Macrolides are highly liposoluble and consequently penetrate well into the lower respiratory tract and its secretions [34]. Plasma protein binding is variable from one macrolide compound to another. At therapeutic concentrations, protein-bound erythromycin accounts for 80–90% of the total drug present in the blood, and the protein-bound fraction is 95% for roxithromycin [34]. Protein binding appears to affect PK parameters, e.g., decreasing distribution volume and renal elimination. It is likely that the lack of plasma protein in PELF plays a role in the higher PELF concentration of a macrolide than serum/plasma. Macrolides reversibly penetrate cells and are highly partitioned into many cells [33]. Particularly in the case of azithromycin, the long serum half-life is partially a result of this intracellular partitioning [33]. Moreover, one of the extravascular delivery mechanisms of macrolides is mediated via phagocytes, which is considered to be an essential distribution pathway during respiratory infections [22,26,27].

As a primary component of the immune defense system, PELF is distributed continuously along the lower respiratory tract and is a potential target of bacterial infection [28]. Compared to plasma, the higher PELF concentration of tulathromycin helps improve antibacterial efficacy during respiratory infections, which is similar to other macrolides [28,35,36]. Macrolides are also inclined to concentrate in bronchoalveolar lavage (BAL) cells and PELF much more than plasma [24,28].

The PK/PD studies based on PELF will aid in understanding the interpretative value of drug concentrations of PELF. Additionally, understanding the relationship between serum/plasma concentrations and concentrations at the infection/target site may be difficult. Can serum/plasma concentrations of a macrolide antibiotic reflect those in the ISF of the target tissue, particularly the respiratory tract? How much difference is there among the serum/plasma concentration, PELF concentration, and pulmonary tissue concentration of a macrolide antibiotic? The following section will address this question.

## 3. Concentrations of Macrolide Antibiotics in Plasma/Serum, Airway Fluid, and Tissues

**Figure 1:**
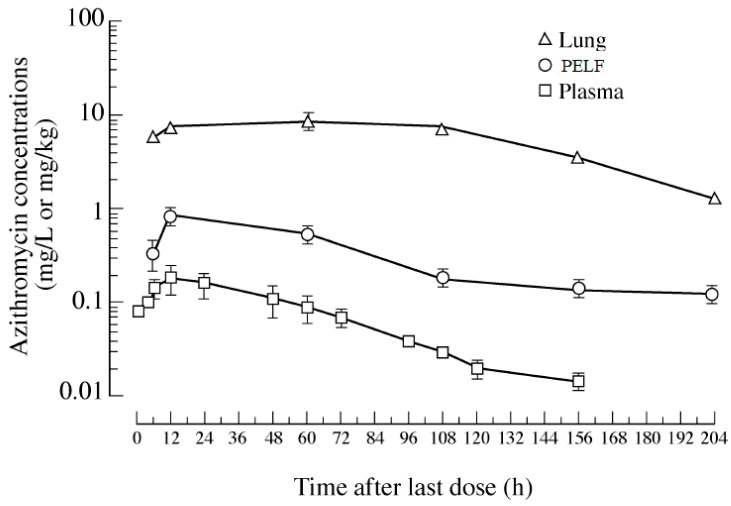
Concentrations vs. time semilogarithmic plot of azithromycin 500 mg per human patient once daily for 3 days in lung tissue, PELF and plasma. (Modified from Danesi et al. [39]). Copyright © 2003, Oxford University Press.

Since the respiratory tract tissue and its epithelial lining fluid contain higher macrolide concentrations than plasma/serum, tremendous research interest has been focused on the area of macrolide concentrations in the airway tissues and associated ISF [12,37]. However, the PK of macrolide antibiotics (e.g., erythromycin, tylosin, azithromycin, clarithromycin, tildipirosin, gamithromycin, tilmicosin, and tulathromycin) in plasma/serum and target tissues, particularly those of the respiratory tract, have not been fully elucidated. Macrolide concentrations are ~10-fold higher in the airway epithelial fluid than in plasma [38]. In a human study, the researchers examined the pharmacokinetics of azithromycin in plasma, lung tissue and PELF in patients after oral administration of 500 mg azithromycin for 3 d. This study examined the pharmacokinetics of azithromycin in plasma, lung tissue, and PELF in patients after the oral administration of 500 mg azithromycin for 3 d. It was found that the plasma azithromycin concentrations were only ~10% and ~1% those of bronchial fluid (BF) and lung, respectively (Figure 1) [39]. The results showed that after azithromycin administration, the drug concentrations were: lung > PELF > plasma. In another two human studies, upon the administration of a single 500 mg oral dose of azithromycin, the tissue concentrations exceeded the minimum inhibitory concentration that inhibited 90% of likely pathogens (MIC_90_), and phagocytic concentrations reached >200 times serum concentrations [40,41]. In another human study, concentrations of clarithromycin and azithromycin in PELF exceeded serum concentrations by 20-fold 24 h after the last dose of drug administration [42]. Telithromycin also has excellent penetration into bronchopulmonary tissues. In humans, concentrations of telithromycin in PEFL exceeded serum concentrations by 12-fold 24 h after the last dose of drug administration [42,43].

**Figure 2:**
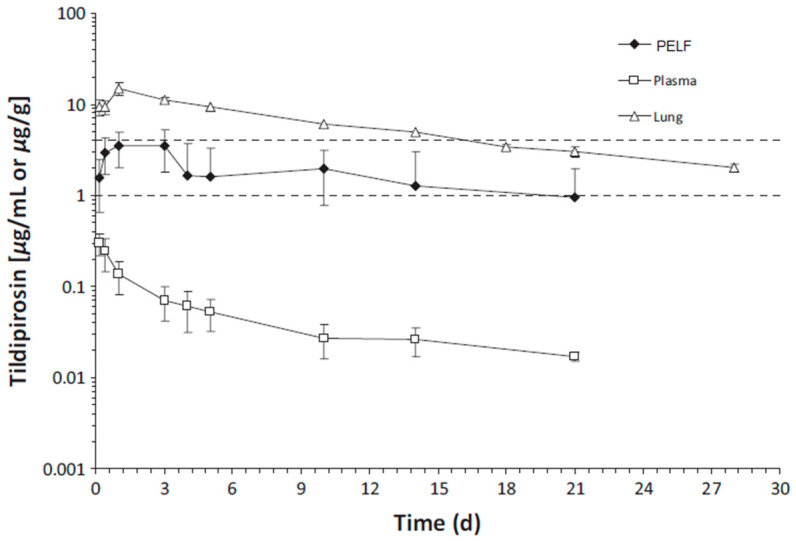
Tildipirosin concentration vs. time (d) in bovine plasma (μg/mL), lung tissue, and PELF (μg/g) from cattle following a single SC administration at 4 mg/kg body weight. The dotted lines represent the MIC_90_ *for Mannheimia haemolytica* and *Pasteurella multocida* (1 µg/mL) and for *Histophilus somni* (4 µg/mL) (Modified from [44]). Copyright © 2012 John Wiley & Sons, Inc.

This phenomenon has been documented in cattle after the administration of tildipirosin as well [13,44,45]. The findings in cattle by Menge et al. [44] showed that the tildipirosin concentrations in lungs collected postmortem (4–240 h after dosing) exceeded those in BF (the ratio of lung/BF concentrations was ~2.5:1) and the ratio of lung/plasma was 27.6:1–214.5:1 (Figure 2). The results showed that after tildipirosin administration, the drug concentrations were: lung > PELF > plasma. The ratio of tildipirosin concentrations of BF/plasma determined from the same animals increased from 5.2:1 (4 h) to 72.3:1 (240 h or 10 days after administration) and then declined to 56.0:1 at the last collection time point of BF and plasma (504 h or 21 days) [44]. The plasma concentrations of tildipirosin were below the MIC_90_ of all three pathogens throughout the study. In contrast, the lung had drug concentrations higher than the MIC_90_ of *H. somni* for 18 d and the MIC_90_ of 2 other pathogens for >28 d. PELF had drug concentrations higher than the MIC_90_ of *H. somni* for 3 d and the MIC_90_ of 2 other pathogens for 21 d [44].

**Figure 3:**
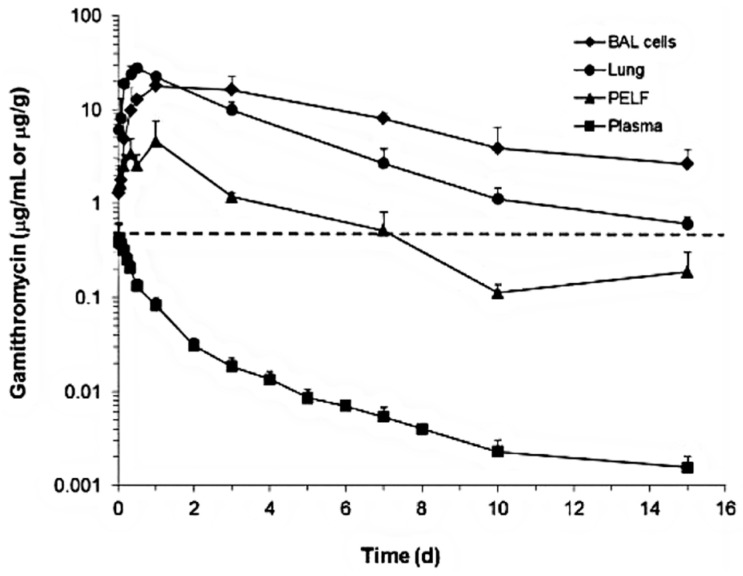
Gamithromycin concentrations in plasma, bronchoalveolar lavage (BAL) cells and pulmonary epithelial lining fluid (PELF) (µg/mL) and lung tissue (µg/g) of healthy Angus calves following gamithromycin administration (6 mg/kg, SC). The dotted line represents the MIC_90_ for *Mannheimia haemolytica* (0.5 µg/mL) [46].

**Figure 4:**
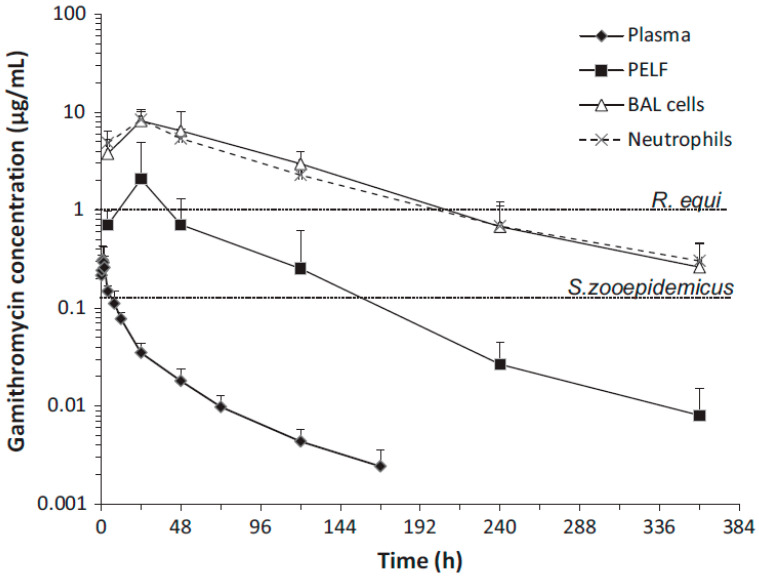
Gamithromycin concentrations in plasma, BAL cells, PELF, and neutrophils of 6 foals following a single IM dose of gamithromycin (6 mg/kg). The dotted horizontal lines represent the MIC_90_ of *Rhodococcus equi* (1 µg/mL) and *Streptococcus zooepidemicus* (0.125 µg/mL) isolates [47]. Copyright © 2012 John Wiley & Sons, Inc.

**Figure 5:**
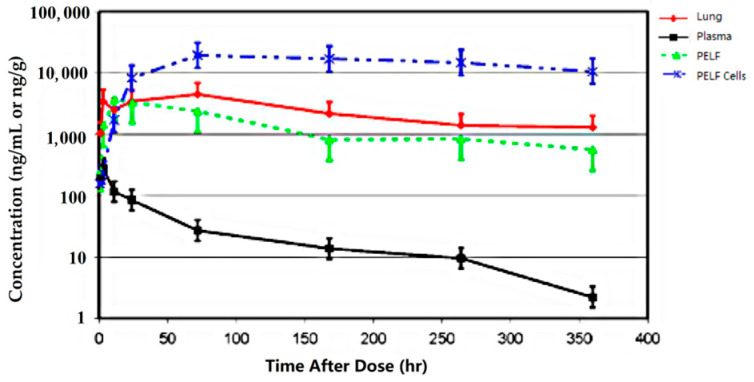
Tulathromycin concentrations for 360 h following a single 2.5 mg/kg IM dose to cattle (Modified from Cox et al. [48]).

Additionally, the concentrations of gamithromycin (Figure 3 and Figure 4) [46,47] and tulathromycin (Figure 5) in bovine lung tissues were also much higher than those of plasma [48]. The results from Giguere, S. et al. (2011) showed that after gamithromycin administration, the drug concentrations were: BAL cells > lung > PELF > plasma [46]. Gamithromycin concentrations in BAL cells and blood neutrophils were 26–732 and 33–563 times higher than concurrent plasma concentrations, respectively. The ratios ranged 4.7:1–127:1 for PELF/plasma, 16:1–650:1 for lung tissue/plasma, and 3.2:1–2135:1 for BAL/plasma [46]. Both BAL cells and lung had drug concentrations above MIC_90_ for >14 d; PELF had drug concentrations higher than MIC_90_ for >6 d; and plasma had drug concentrations below MIC_90_ throughout the study [46]. The results from Berghaus, L.J. et al. (2012) showed that after gamithromycin administration, the drug concentrations were: BAL cells = neutrophils > PELF > plasma [47]. Both BAL cells and neutrophils had drug concentrations above the MIC_90_ of both pathogens for >336 h (14 d); PELF had drug concentrations higher than the MIC_90_ of *R. equi* for 48 h (2 d) and higher than the MIC_90_ of *S. zooepidemicus* for >144 h (6 d). Plasma had drug concentrations below the MIC_90_ of both pathogens throughout the study [47]. The plasma concentrations of tulathromycin in cattle were substantially lower than the lung tissue concentrations, with AUC_0-360_ (area under the plasma concentration–time curve) values for PELF, PELF cells, and lung homogenate of cattle (Figure 5) [48]. The results showed that after tulathromycin administration, the drug concentrations were: PELF cells > lungs > PELF > plasma. Likewise, tulathromycin concentrations in PELF were ~10 times higher than plasma concentrations with the IV/IM route in horses [49]. The AUC_0–17_ days in BF and PELF were 223 and 90 times, respectively, the corresponding values of plasma. The PELF and BF concentration profiles of tulathromycin revealed that there was disparity in the local PK of tulathromycin at various anatomical structures of the lung. Therefore, drug concentrations in PELF may not be a precise alternative of drug concentrations for different pulmonary compartments [50]. The equilibrium between plasma–BF and plasma–PELF does not occur instantaneously, and the concentration–time profiles in both compartments are different [51,52]. The PELF and BF drug concentrations are mainly affected by the alveolar area and bronchial section, respectively. The larger blood flow and surface area of the airway compartment compared with the alveolar and bronchial areas may account for, at least partially, the differences in tulathromycin profiles at those two lung compartments [50].

**Figure 6:**
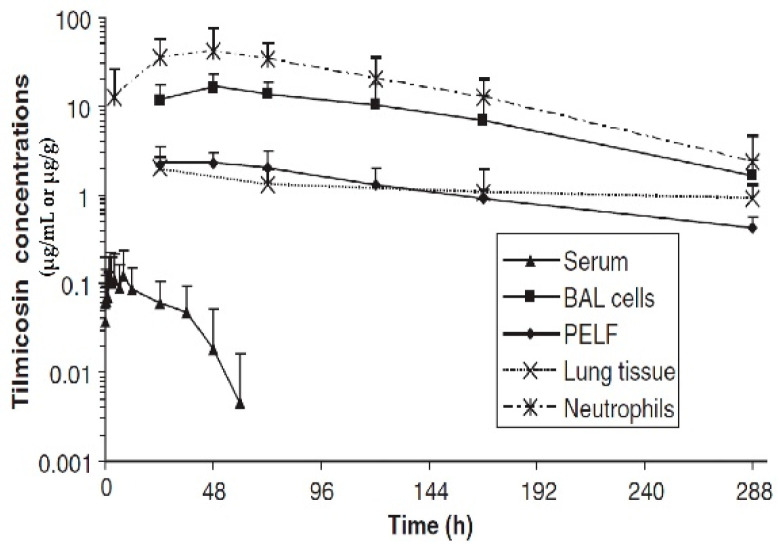
Tilmicosin concentrations in serum, bronchoalveolar lavage (BAL) cells, pulmonary epithelial lining fluid (PELF) (µg/mL), and lung tissue (µg/g) of 7 foals following a single IM dose of tilmicosin (10 mg/kg) [53]. Copyright © 2006 John Wiley & Sons, Inc.

Similarly, the C_max_ and AUC of tilmicosin in foals were higher for PELF than for serum (Figure 6) [53]. The results showed that after tilmicosin administration, the drug concentrations were: lung > BAL cells > PELF = neutrophils > serum. Following intragastric administration, the ratios of PELF/plasma telithromycin concentrations in foals were 5.89:1 and 5.64:1 at 4 and 24 h, respectively [54].

The summary of the ratio thereof and the selected PK parameters based on the mean lung/PELF and plasma concentrations of several macrolide antimicrobials are shown in Table 1.

Because macrolide antibiotics achieve concentrations in lung tissue and ISF that are many times the plasma/serum concentrations, the plasma-free drug concentrations may never reach the lung ISF level. In addition, the vast majority of bacterial pathogens are found in the ISF of the respiratory tract. These phenomena suggest that the macrolide concentration of the airway ISF is a more appropriate predictor of antibacterial activity than either lung tissue or plasma/serum concentrations. Furthermore, pathophysiological responses to infection, including increased phagocyte trafficking to the ISF and vascular permeability, may facilitate local drug delivery [56]. Therefore, the plasma/serum concentration of macrolide antibiotics is not an ideal indicator of in vivo efficacy against pulmonary bacterial infections [57]. It has been stated that the concentrations of lung tissues and ISF are more relevant than those of plasma/serum in determining the outcome of the treatment with a macrolide antibiotic [58]. On the other hand, Toutain et al. had a different opinion in an article published in 2017 [59]. In this study, they found that the ISF collected from the subcutaneous tissue after tulathromycin administration in calves had lower antibiotic concentrations than plasma. Based on these results, they recommended “standard PK/PD concepts can be applied to determine a regimen for a macrolide”. However, the ISF of different tissues has different macrolide concentrations; lungs have higher concentrations than muscle, fat, and skin [20,21,22]. In addition, pulmonary ISF concentrations of a macrolide are much higher than those of plasma [38]. It is possible that some of the reported high PELF concentrations of macrolides are due to the contamination by lysed airway lining cells during bronchoalveolar lavage [60]. However, much higher macrolide concentrations were found in PELF than those of serum/plasma using microdialysis, a minimally invasive ISF collection technique [51,58]. Nevertheless, higher PELF concentrations of macrolides can be collected without significant contamination by airway lining cells. Therefore, we suggest the PK/PD paradigm using PELF/BF concentrations as input values be applied to determine the optimal dose regimen for a macrolide antibiotic in the treatment of pulmonary bacterial infections.

How do pharmaceutical companies determine the optimal dose regimen of macrolide antibiotics? The determination of the optimal dose regimen for antibiotics has a clinical cure as its aim. It is widely acceptable that the optimal dosage for clinical use can only be established in a clinical setting using a dose titration approach [59]. The results of drug development studies have demonstrated that the pulmonary ISF concentrations of a number of macrolide antibiotics (e.g., tildipirosin (NADA141-334), gamithromycin (NADA 141-328), tilmicosin (NADA141-361), and tulathromycin (NADA 141-244)) in animals are consistently higher than the plasma concentrations of these antibiotics. In addition, following the designated dose regimen by the pharmaceutical company, the tissue ISF levels of macrolide antibiotics are maintained above the MICs of major pathogens for an extended period of time (see the aforementioned NADAs); this would justify the clinical use of the designated dose regimen for the macrolide antibiotic. However, to the best of our knowledge, the ISF-based PK paradigm has not been used for the determination of PD as well as optimal dose regimen for macrolide antibiotics in clinical practice. In light of the challenges to the applicability of the PK/PD paradigm to macrolides, the use of lung ISF-derived PK data should improve the optimal dose regimen determination of a macrolide antibiotic for the treatment of lower respiratory tract bacterial infection.

## 4. Approaches for Airway ISF Collection

Since the ISF of the respiratory tract is the target site for most pathogenic bacteria and both BF and PELF contain much higher macrolide concentrations than plasma/serum, many more studies are warranted to evaluate the relevance of macrolide concentrations in the ISF of the respiratory tract. BF and PELF have been considered the principal target sites of the bacterial infections of the respiratory tract. The methods employed for collecting BF and PELF include the direct collection of BF, the pulmonary tissue homogenization technique, the BAL technique, and the microdialysis technique [60]. The tissue homogenization technique is no longer considered a valid technique for collecting ISF because the supernatant of the homogenate harvested after centrifugation produces a sample containing both intracellular and extracellular fluid. This approach overestimates drug concentrations, particularly those of macrolides in the extracellular space, because the intracellular macrolide concentration is much higher than that of ISF [60]. In contrast, the use of BAL to collect BF and PELF will avoid the contamination associated with the tissue homogenization technique. However, the BAL technique including the use of bronchoscopy is associated with technical complexity [60]. Different investigators may have different results with the main variations being the dwell time, the aspiration pressure (high pressure may damage epithelial linings), the volume of fluid injected, and the number of BAL aspirates collected [61]. To circumvent the problem associated with the BAL technique, a number of novel techniques including microdialysis can be used to confirm drug concentrations in ISF [62]. Free drug concentrations in ISF can be quantified over time using microdialysis; this technique is the gold standard in both human medicine [63] and veterinary medicine [64]. The technique of microdialysis enables the monitoring of a variety of molecules in ISF [65,66]. Microdialysis is a minimally invasive sampling technique employing membrane probes implanted in the tissue of interest in awake and freely moving subjects [67]. Microdialysis is a technique that uses a probe with a semipermeable membrane to insert into a tissue of interest. The probe is constantly flushed with a physiological buffer solution that allows the drug under study to leak out of the membrane and to be analyzed in the solution. In vivo microdialysis is a common in vivo method that measures the unbound (free) drug in the extracellular fluid of various tissues. The method uses a porous fiber membrane that allows the free drug molecules to cross the membrane along the concentration gradient. Microdialysis is performed by placing the probe made of a hollow fiber membrane into the extracellular fluid of the tissue where the free drug is to be measured. The probe has two tubes attached: one for the inlet and the other for the outlet where a physiological buffer flows through. As the buffer flows, the free drug molecules leak out of the pores into the outlet tube, and thus they can be detected [68]. The details of this technique are described by others [68].

## 5. Conclusions

Although the change in plasma/serum concentration–time and the change in airway ISF–concentration–time of macrolide antibiotics are proportional, macrolide antibiotics in plasma/serum do not reflect the antibacterial activity of the airway ISF. Thus, if the ISF concentrations of a macrolide antibiotic are efficiently and precisely collected using the least invasive method, e.g., microdialysis, practical PK/PD parameters can be obtained for such a study. Nevertheless, the PK data of airway ISF, the site of bacterial infection, are more important in setting the optimal dose regimen of a macrolide than the PK data of plasma/serum.

## Figures and Tables

**Table 1:** Summary of selected PK parameters determined based on mean macrolide antibiotic concentrations in plasma, lung, and pulmonary epithelial lining fluid (PELF).

Drug	Species	Regimen	PK Parameter	Lung	Plasma	PELF	Ratio of Lung/Plasma	Ratio of PELF/Plasma	References
**Tidipirosin**	Cattle	SC 4 mg/kg BW	**PK Parameter (units)**						[44]
			AUC_last_ (µg·h/mL)	3846.735	24.208	882.45	159	37	
			AUC_inf_ (µg·h/mL)	4497.563	28.973	1233.75	155	43	
			T_1/2_ (h)	242	216	267	1	1	
**Gamithromycin**	Cattle	SC 6 mg/kg BW	**PK Parameter (units)**						[46]
			AUC_last_ (µg·h/mL)	2154	7.82	334	275.4v	42.7	
			AUC_inf_ (µg·h/mL)	2235	7.95	348	281.1	43.7	
			C_max_ (µg·h/mL)	27.8	0.433	4.61	64.6	3.2	
			T_max_ (h)	12.01	1.00	24.0	12.01	24	
			T_1/2_ (h)	93.0	62.0	-	1.5	NR	
**Tulathromycin**	Cattle	SC 2.5 mg/kg BW	**PK Parameter (units)**						[48]
			AUC0-360 (µg·h/mL)	867	9.26	492	93.6	53.13	
			C_max_ (ng/mL)	4510	277	3730	16.28	35.13	
			T_max_ (h)	72	3	11	24	3.67	
			T_1/2_ (h)	279	64	330	4.35	5.15	
**Tulathromycin**	Pig	IM 2.5 mg/kg BW	**PK Parameter (units)**						[50,51,52]
			AUC_last_ (ng·h/mL)	NR	5670	435000	NR	76.7	
			AUC_inf_ (ng·h/mL)	NR	5650	473500	NR	83.8	
			C_max_ (ng/mL)	NR	458	5235	NR	11.4	
			T_1/2_ (h)	NR	55.82	97.65	NR	1.75	
**Azithromycin**	human	PO 500 mg/once daily for 3 days	**PK Parameter (units)**						[39]
			AUClast (mg·h/L)	1245.4	11.62	70.29	107.2	6.04	
			Cmax (mg/L)	8.93	0.18	0.83	5.55	4.6	
**Azithromycin**	Foals	IG 10 mg/kg BW	**PK Parameter (units)**						[55]
			AUCinf (µg·h/mL)	NR	7.70	247	NR	35.2	
			C_max_ (µg/mL)	NR	0.83	10.00	NR	5.4	
			T_1/2_ (h)	NR	25.7	34.8	NR	1.35	
**Clarithromycin**	Foals	IG 10 mg/kg BW	**PK Parameter (units)**						[55]
			AUC_last_ (mg·h/L)	NR	4.76	629	NR	132.14	
			C_max_ (mg/L)	NR	0.94	48.96	NR	52	
**Tilmicosin**	Foals	IM 10 mg/kg BW	**PK Parameter (units)**						[53]
			AUC_inf_ (µg·h/mL)	711	5.76	461	123.4	80	
			C_max_ (µg/mL)	1.90	0.19	2.91	10	15.3	
			T_1/2_ (h)	193.3	18.4	73.1	10.5	3.97	

PO = Oral route; SC = subcutaneous route; IV = intravenous route6; IG = intragastric route; BW = body weight; NR = not reported; Conc = concentration AUC_last_, area under the plasma concentration–time curve from time 0 to the last quantifiable timepoint (t_last_); AUC_inf_, area under the plasma concentration–time curve from time 0 to infinity; C_max_, maximum plasma concentration; T_max_, time of occurrence of C_max_; t_1/2_, elimination half-life.4. Which Matrix (Lung Tissue, ISF, or Plasma/Serum) Plays a Vital Role in Determining Macrolide Antibiotic Dose Regime?

## Data Availability

Not applicable.

## Abbreviations

BF	bronchial fluid
BAL	bronchoalveolar lavage cells
ISF	interstitial fluid
MIC	minimum inhibitory concentrations
PELF	pulmonary epithelial lining fluid
PK	pharmacokinetics
PD	pharmacodynamics
